# Roles of the kidney in the formation, remodeling and repair of bone

**DOI:** 10.1007/s40620-016-0284-7

**Published:** 2016-03-04

**Authors:** Kai Wei, Zhiwei Yin, Yuansheng Xie

**Affiliations:** 1 Department of Nephrology, Chinese PLA General Hospital, Chinese PLA Institute of Nephrology, State Key Laboratory of Kidney Diseases, National Clinical Research Center for Kidney Diseases, 28 Fuxing Road, Beijing, 100853 People’s Republic of China; 2 Medical College, NanKai University, Tianjin, 300071 People’s Republic of China

**Keywords:** Kidney, Bone, 1,25(OH)_2_D_3_, Klotho, Bone morphogenetic protein-7, Erythropoietin

## Abstract

The relationship between the kidney and bone is highly complex, and the kidney plays an important role in the regulation of bone development and metabolism. The kidney is the major organ involved in the regulation of calcium and phosphate homeostasis, which is essential for bone mineralization and development. Many substances synthesized by the kidney, such as 1,25(OH)_2_D_3_, Klotho, bone morphogenetic protein-7, and erythropoietin, are involved in different stages of bone formation, remodeling and repair. In addition, some cytokines which can be affected by the kidney, such as osteoprotegerin, sclerostin, fibroblast growth factor -23 and parathyroid hormone, also play important roles in bone metabolism. In this paper, we summarize the possible effects of these kidney-related cytokines on bone and their possible mechanisms. Most of these cytokines can interact with one another, constituting an intricate network between the kidney and bone. Therefore, kidney diseases should be considered among patients presenting with osteodystrophy and disturbances in bone and mineral metabolism, and treatment for renal dysfunction may accelerate their recovery.

## Introduction

The relationship between the kidney and bone is a field that has been explored for a very long time. Over 2000 years ago, the earliest Chinese medical literature ‘Huangdi Neijing’ systematically expounded the theory “shen zhu gu” (kidney controls bone) from the perspective of traditional Chinese medicine. In 1943, two Chinese physicians introduced the term “renal osteodystrophy” to describe the cases of osseous disorder associated with renal insufficiency [1]. All bone abnormalities related to chronic kidney disease (CKD) ultimately lead to an increased risk of fracture, which has become an important cause of morbidity and decreased quality of life [2]. Hence, normal function of kidney is important for bone health, and to illustrate the potential relationships between kidney and bone becomes an urgent issue. In this review, we recapitulate the possible links between the kidney and bone with a main focus on the role of the kidney.

## The kidney is the major organ for homeostasis of calcium and phosphate

The homeostasis of calcium and phosphate is complicated. In calcium and phosphate balance, more than 97 % of calcium and 80 % of phosphate filtered in the kidney are reabsorbed at different segments of the tubules, and their homeostasis can be severely affected in kidney diseases [3, 4]. The majority of the calcium and phosphate in our body resides in bone, and these two elements are critical for the normal structure and function of bone [5]. Calcium and phosphate are the main components of bone minerals. Beyond that, they also have some biological effects. Calcium can directly stimulate osteoblast formation while inhibiting osteoclast formation via calcium-sensitive receptor (CaSR) dependent or independent pathways [6, 7]. However, phosphate can directly promote osteoclast apoptosis and inhibit its differentiation by affecting receptor activator of nuclear factor-κB (RANK)-RANK ligand (RANKL) signaling and osteoprotegerin (OPG) [8, 9]. This indicates that the kidney may affect the structure and function of bone by regulating the homeostasis of calcium and phosphate.

## The kidney regulates bone health by generating activated vitamin D

1,25(OH)_2_D_3_ is the activated pattern of vitamin D, and the circulating 1,25(OH)_2_D_3_ (calcitriol) is mainly produced in the proximal renal tubules under the hydroxylation of 1α-hydroxylase [1α(OH)ase], which is encoded by *cyp27b1* [10]. It regulates the homeostasis of calcium and phosphate, and bone development and repair by binding vitamin D receptor (VDR) located in the intestine, kidney and bone.

1,25(OH)_2_D_3_ promotes calcium and phosphate uptake and reabsorption by increasing the expression of their transport proteins in enterocytes and renal tubule cells [3, 11]. Apart from that, direct effects on bone are also observed. Chondrocyte-specific *vdr* inactivation in mice shows that 1,25(OH)_2_D_3_ controls vascular invasion and osteoclast formation by increasing vascular endothelial growth factor (VEGF) and RANKL [12]. For osteoblast, 1,25(OH)_2_D_3_ affects the synthesis of collagen I and expression of alkaline phosphatase (ALP), osteocalcin and osteopontin [13, 14], stimulates bone matrix mineralization via accelerating the production of mature micro vesicles and modulates the bone microenvironment by regulating the osteoblastic niche [15, 16]. For osteoclast, 1,25(OH)_2_D_3_ plays bidirectional roles. On the one hand, it stimulates osteoclastogenesis by increasing the expression of RANKL on chondrocyte and osteoclast [12, 17]. On the other hand, in osteoclast precursors, 1,25(OH)_2_D_3_ directly suppresses the expression of RANK via down-regulation of c-Fms, inhibits key regulators of osteoclast formation, c-Fos and NFATc1, and increases its inhibitor, CCAAT enhancer-binding proteins [18–21]. Therefore the comprehensive effects of 1,25(OH)_2_D_3_ on osteoclast, osteoclastogenesis and bone resorption need to be further investigated.

**Fig. 1:**
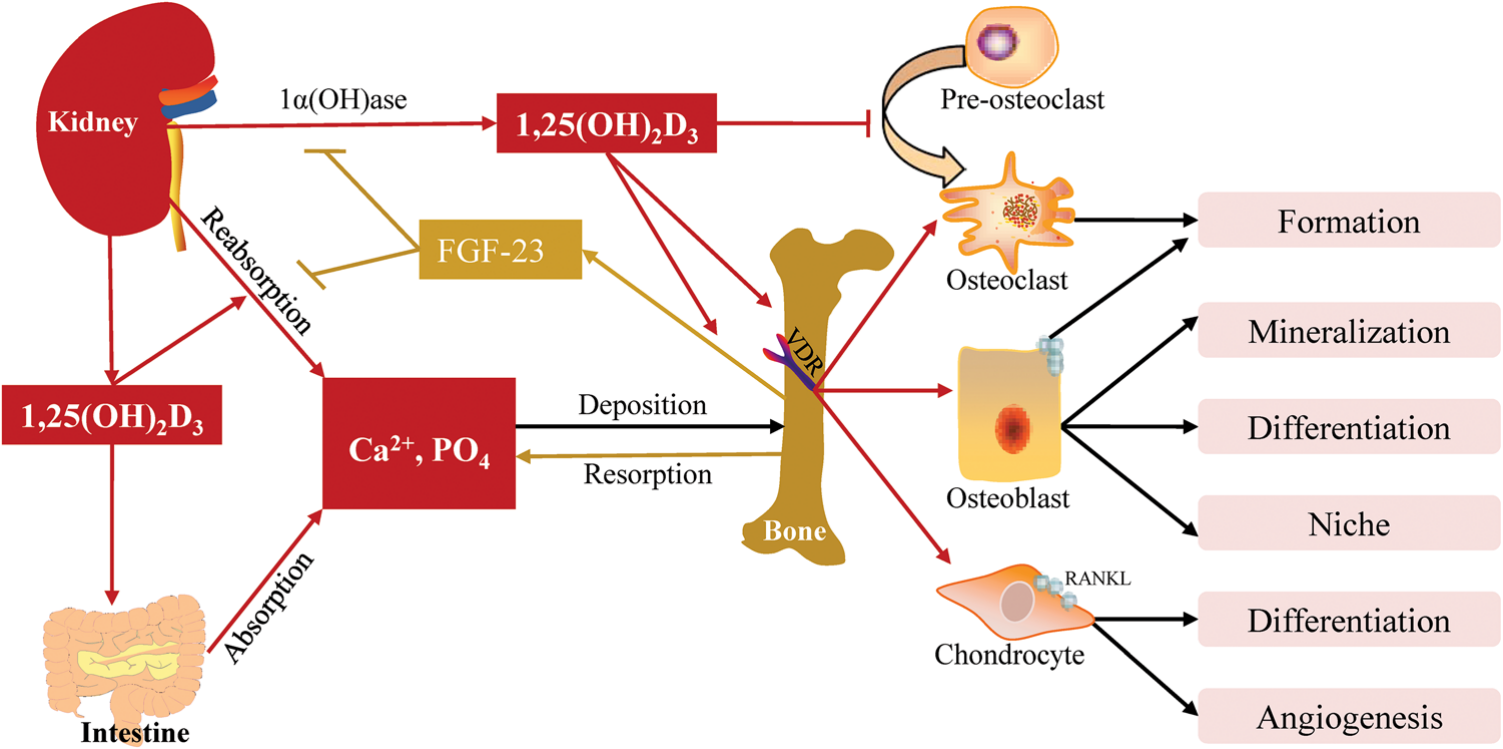
Actions of 1,25(OH)_2_D_3_ on calcium and phosphate homeostasis and bone development. 1,25(OH)_2_D_3_ synthesized by the kidney promotes the absorption and reabsorption of calcium and phosphate in the intestine and kidney, stimulates FGF-23 production in bone, which inhibits 1,25(OH)_2_D_3_ synthesis and phosphate reabsorption in kidney. 1,25(OH)_2_D_3_ exerts osteogenic effects on osteoblasts and chondrocytes and inhibits osteoclast differentiation while promoting its maturation by up-regulating RANKL expression in osteoblasts. : produce or promote;: inhibit, 1α-(OH)ase: 1α-hydroxylase, Ca^2+^: calcium, FGF: fibroblast growth factor, PO_4_: phosphate, RANKL: receptor activator of nuclear factor NF-κβ ligand, VDR: vitamin D receptor

In addition, 1,25(OH)_2_D_3_ can stimulate fibroblast growth factor (FGF)-23 secretion in osteocytes via binding vitamin D response element (VDRE) [22]. Reversely, FGF-23 suppresses 1,25(OH)_2_D_3_ levels via its effects on the kidney to stimulate CYP24A1-mediated degradation and suppress 1α(OH)ase-mediated production [23]. The negative feedback loop between them plays an important role in the crosstalk between the kidney and bone (Fig. 1).

## The kidney maintains bone formation and remodeling by producing Klotho

Klotho is identified as an “aging suppressor” protein, which is primarily expressed in renal distal convoluted tubules [24]. It can be divided into membrane-binding and soluble forms. Membrane-binding Klotho forms a complex with FGF receptors (FGFRs) and functions as an essential co-receptor for FGF-23 [25]. The Klotho/FGFR/FGF-23 complex, except for its effect on 1,25(OH)_2_D_3_ mentioned above, can suppress sodium-phosphate (NaPi) co-transport activity in kidney and reduce phosphate reabsorption [26]. Furthermore, as for proximal tubule epithelial cells (PTEC), exposure to both FGF-23 and Klotho initiates Ras and phosphatidylinositol 3-kinase (PI3K) signaling pathways manifested by up-regulation in phosphorylation of ERK1/2, p38, JNK, AKT, IkB and GSK-3β. Combined application of FGF-23 and Klotho rescues high 1,25(OH)_2_D_3_-induced apoptosis of PTEC, while PI3K inhibitor prevents the effect of FGF-23 and Klotho [27]. Therefore, both Ras and PI3K signaling pathways may be involved in the crosstalk between the kidney and bone.

Different from membrane-binding Klotho, soluble Klotho can be released into the circulation and act on remote organs in FGF-23 dependent and independent ways. Soluble Klotho interacts with the FGFRs in osteoblast and facilitates FGF-23 induced proliferation and inhibition of mineralization [28]. In addition, it can exert phosphaturic effects independently. In normal and *Fgf23* ^−/−^ mice, soluble Klotho is able to inactivate NaPi-IIb in the intestine and NaPi-IIa in the proximal renal tubules to reduce phosphate absorption and reabsorption [24]. Furthermore, it can also activate calcium channels transient receptor potential vanilloid receptor (TRPV) 5/6 and conserve serum calcium and reduce calciuria [29, 30]. Since soluble Klotho can inhibit IGF-1, Wnt and transforming growth factor-β (TGF-β) signaling pathways in aging and in certain cancers [31], whether these pathways are involved in the Klotho-induced regulation of bone formation still needs to be investigated.

**Fig. 2:**
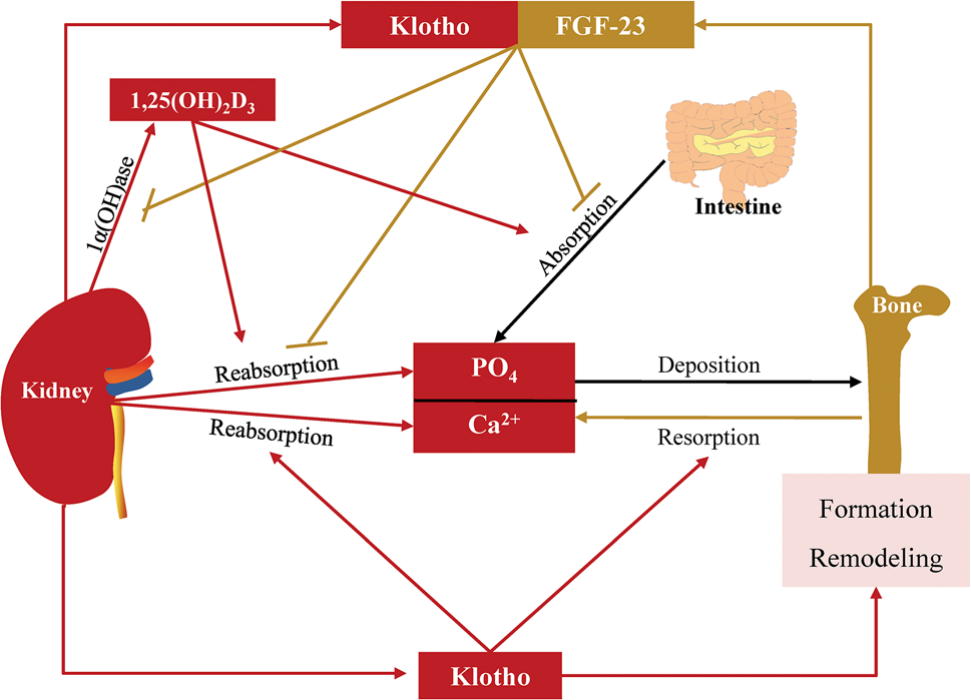
Actions of Klotho on calcium and phosphate homeostasis and bone. The complex of Klotho and FGF-23 inhibits 1,25(OH)_2_D_3_ synthesis and phosphate reabsorption in the kidney. Soluble Klotho stimulates calcium reabsorption and resorption in the kidney and bone, inhibits calcium and phosphate absorption in intestine and regulates bone formation and remodeling. : produce or promote, : inhibit, 1α(OH)ase: 1α-hydroxylase, Ca^2+^: calcium, FGF: fibroblast growth factor, PO_4_: phosphate

In addition, direct effects of Klotho on bone formation and remodeling are also observed. Klotho deficient mice show low bone formation and bone resorption activities, which result in osteopenia [32]. Histological studies show that the expression of osteocalcin and dentinmatrix protein-1 (DMP-1) were weak and uneven in the tibiae matrix of the *Klotho* ^−/−^ mice, and matrix Gla protein (MGP) expressed in the cartilage cores and bone surfaces, which were different from wild type mice whose MGP mainly expressed at the junction between cartilage and bone [32]. Furthermore, the *Klotho* ^−/−^ mice fail to have formed compact bone in lumbars manifested as abundant osteocytes, pyknotic osteocytes and random empty lacunae [33]. Although osteoblasts from these mice proliferate normally in vitro, their ability to produce ALP and to mineralize extracellular matrix is reduced. Moreover, the low bone resorption activities related to the impaired osteoclastogenesis appears to be associated with up-regulation of OPG synthesis, which can suppress osteoclast differentiation [34, 35] (Fig. 2). However, Klotho overexpression can also result in reduced bone mineral content, expanded growth palates and fracture [36]. Therefore, kidney derived Klotho acts on bone either independently or in cooperation with bone derived FGF-23, and both the pathological increase and decrease of Klotho can cause disturbed bone development and metabolism; hence, a desired range of circulating Klotho is critical for bone health.

## The kidney modulates bone development by producing bone morphogenetic protein-7

Bone morphogenetic protein-7 (BMP-7), a member of the TGF-β superfamily, was originally isolated from demineralized bone based on its ability to induce new bone formation. The kidney has been identified as the major site for BMP-7 synthesis during embryonal and postnatal development [37]. As a determinant of the embryogenesis of bone, the effects of BMP-7 on bone have been widely investigated in different types of animal models. It has been demonstrated that BMP-7 can induce bone formation, enhance incomplete fracture and bone defects, even ameliorate osteonecrosis, osteoarthritis and intervertebral disc degeneration [38–40]. Mechanisms underlying the beneficial effects of BMP-7 on bone are sophisticated. For chondrocytes, BMP-7 can promote chondrocyte differentiation from bone marrow mesenchymal stem cells (BMMSCs) and exert anabolic effects by stimulating synthesis of extracellular matrix proteins [41]. In addition, BMP-7 can exert anti-catabolic effects on chondrocytes by blocking metalloproteinase (MMP)-1, MMP-13 and a disintegrin and metalloproteinase with thrombospondin motifs (ADAMTMS) that destroy the extracellular matrix and cartilage [40, 42]. In some cases, BMP-7 can also promote chondrocyte maturation manifested by hypertrophy and increased expression of ALP [43]. For osteoblasts, BMP-7 is capable of inducing its differentiation from BMMSCs by expressing osteoblast differentiation markers, such as ALP and Runx2, stimulating the proliferation of mature osteoblasts, inducing collagen synthesis and enhancing its activity by expressing osteocalcin [41, 44, 45]. The potential mechanisms involved in the above processes are far more complicated. As members of the TGF-β superfamily, BMP-7 can activate canonical signaling including Smad1/4/5/8 and non-canonical TAK1/MKK/p38 signaling. In addition, the interplay between BMPs and Notch, Hh, FGF as well as Wnt signaling also plays a very important role in osteoblast and bone [46]. However, whether those signaling pathways are involved in the BMP-7 induced bone development still needs to be investigated.

**Fig. 3:**
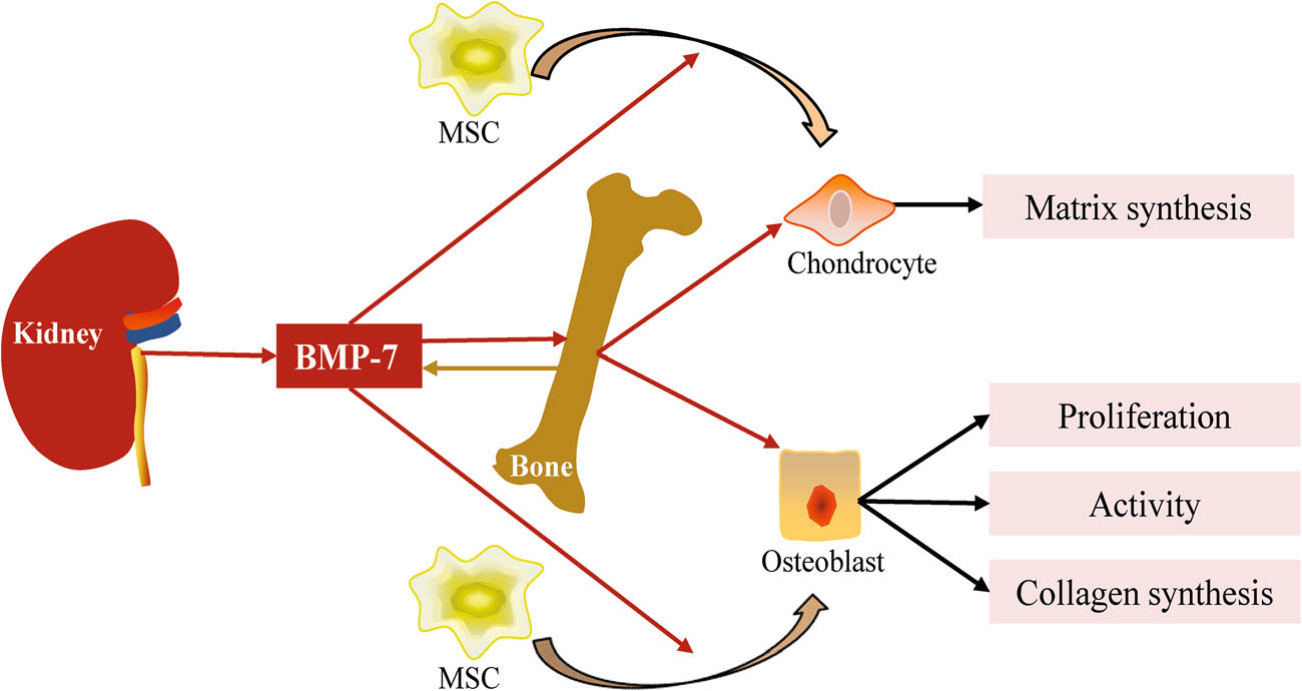
Actions of BMP-7 on bone. Kidney produces BMP-7. BMP-7 stimulates chondrocyte and osteoblast differentiation from MSC; it also induces their osteogenesis. : produce or promote, : inhibit, BMP: bone morphogenetic protein, MSC: mesenchymal stem cell

There is a relatively low level of BMP-7 production in bone, and it seems not to be required for skeletal homeostasis because conditional deletion of BMP-7 from the limb skeleton has no effect on postnatal limb growth, articular cartilage formation, maintenance of bone mass, or fracture healing [47]. The results above suggest that extra-skeletal derived BMP-7 may be more important in bone formation and growth. The kidney is the major site of BMP-7 synthesis, and BMP-7 produced in the kidney is constantly released into the circulation, functioning at distant sites in a hormone-like manner [48]. Furthermore, in rat models of osteodystrophy due to renal mass ablation, administration of recombinant BMP-7 can successfully inhibit the bone disorder [49] (Fig. 3). All of the above indicate that BMP-7, which is required for normal bone development and metabolism, originates from the kidney.

## The kidney promotes bone development and fracture healing by synthesizing erythropoietin

Erythropoietin (EPO) is a 30.4kD glycoprotein and class I cytokine that is characterized by its role in the regulation of red blood cell production in bone marrow due to its expression of EPO receptor (EPO-R) [50]. Approximately 90 % of systemic EPO in adults is produced by peritubular interstitial fibroblasts in the renal cortex and outer medulla of the kidney [51]. In recent years, with the identification of non-hematopoietic EPO-R and CD131, the non-hematopoietic effects of EPO have begun to be investigated.

**Fig. 4:**
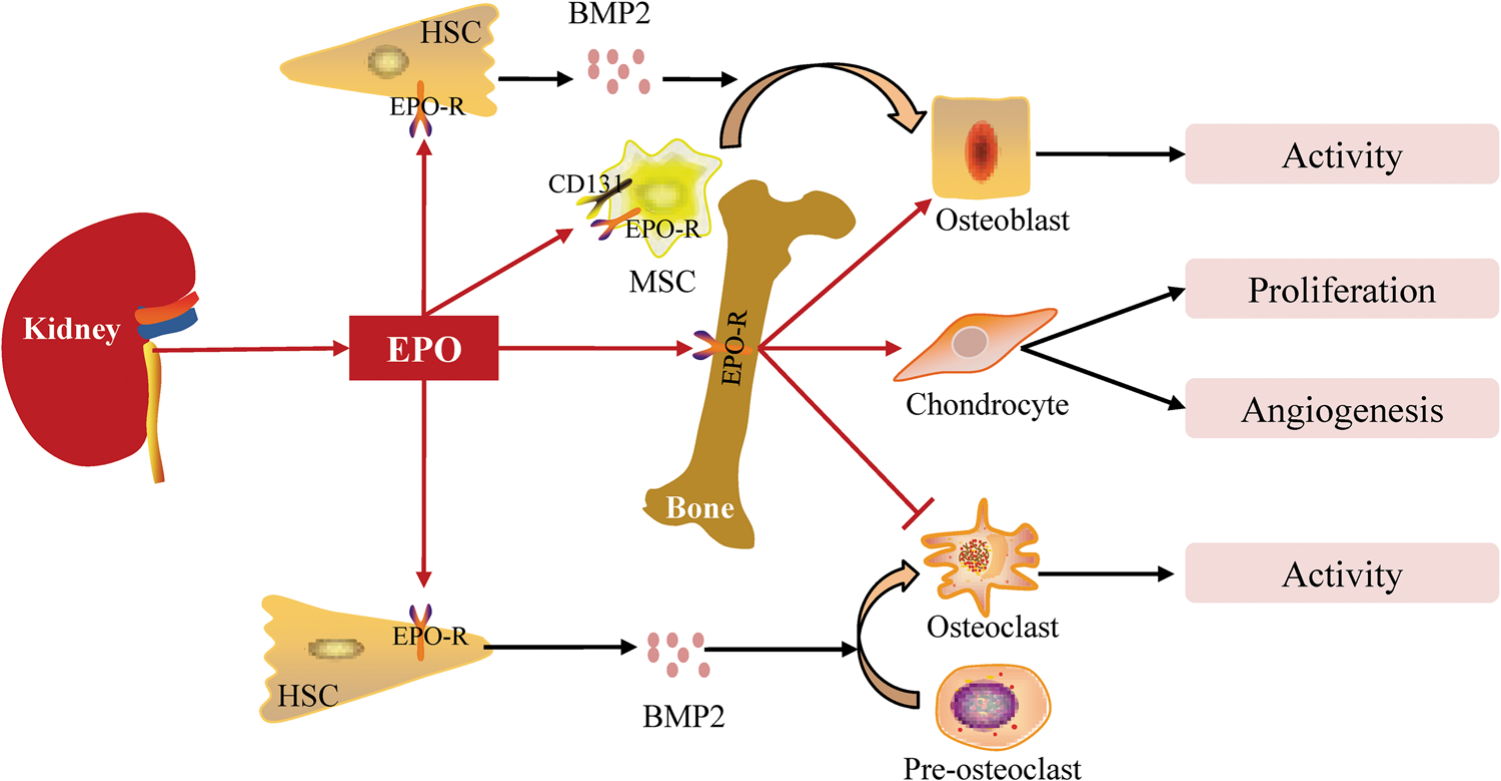
Actions of EPO on bone. EPO produced by the kidney promotes osteoblast activity, stimulates chondrocyte proliferation and angiogenesis and decreases osteoclast activity by binding EPO-R. EPO stimulates osteoblast and osteoclast differentiation from their precursors by up-regulating BMP2 expression by HSC and stimulates osteoblast differentiation from MSC by binding EPO-R or CD131. : produce or promote, : inhibit, BMP: bone morphogenetic proteins, EPO: erythropoietin, EPO-R: erythropoietin receptor, HSC: hematopoietic stem cell, MSC: mesenchymal stem cell

Recent studies indicate that EPO plays an important role in bone formation, fracture healing and intervertebral disc degeneration. In fracture models of mice, it has been found that the terminally differentiated chondrocytes within the callus expressed EPO-R and that administration of recombined EPO is capable of stimulating endochondral ossification, cell proliferation and VEGF-mediated angiogenesis [52]. A rabbit model of autograft posterolateral spinal fusion also shows that systemic EPO administration can increase bone volume and neovascularization [53]. First, EPO can increase BMP2 expression by hematopoietic stem cells (HSCs) through the JAK2/Stat3 signaling pathway. Then, EPO can induce osteoblasts differentiation from BMMSCs in vitro and bone formation in vivo either directly or indirectly by the expression of BMP-2 by HSCs [54]. In addition, EPO can increase osteoclast numbers, but its effects on osteoclast activity still need to be investigated [55]. Further investigations demonstrate that the osteogenic and osteoclastogenesis effects of EPO can be mediated by three intracellular signaling pathways: mammalian target of rapamycin (mTOR), JAK2 and PI3K [56] (Fig. 4). Given that EPO is a renal hormone, the kidney likely plays an important role in bone formation and fracture healing via EPO.

## Other factors that are involved in the regulation of bone remodeling

Osteoprotegerin, secreted by osteoblast lineage cells, belongs to the tumor necrosis factor (TNF) family of receptors [57]. Known as a decoy receptor for the pro-osteoclastic cytokine RANKL, OPG can inhibit osteoclast differentiation and activation. Clinical studies indicate that the levels of OPG are increased in patients with CKD stages I through V and that serum levels of OPG correlate with serum creatinine levels and have a reciprocal relationship to creatinine clearance over a 24-h period [58]. Furthermore, OPG levels drop to laboratory norms after renal transplantation in parallel with renal function restoration [59]. Thus, the kidney is recognized as the major site for clearance of OPG.

Sclerostin, another bone formation regulator, is secreted by osteocytes. As an antagonist for BMPs and Wnt signaling, sclerostin can modulate the activity of osteoblasts by reducing ALP activity, synthesis of type I collagen and mineralization [60, 61]. In addition, sclerostin can also promote osteoblast apoptosis in vitro [62]. Recent studies show that serum sclerostin levels are increased in patients with CKD and are negatively correlated with estimated glomerular filtration rate (eGFR) following correction for age and gender. Moreover, the elevated serum sclerostin levels reduce rapidly in parallel with the improvement of renal function, which suggests that the kidney probably participates in sclerostin clearance [63, 64]. Therefore, the kidney can also regulate bone formation by clearing some bone regulators.

In addition, parathyroid hormone (PTH) and FGF-23 are key regulators in mediating bone and mineral abnormalities caused by CKD. Both PTH and FGF-23 begin to increase in the early stage of CKD, when the eGFR drops below 60 ml/min per 1.73 m^2^ [65]. Early control PTH and FGF-23 can improve bone and mineral disturbances in CKD effectively [66, 67]. PTH, on the one hand, can increase the number and activity of osteoclasts by regulating the expression of RANKL and OPG [68]; on the other hand, it can suppress chondrocyte and osteoblast differentiation manifested as reduced mineralization, and decreased expression of Runx2, ALP, procollagen I and osteocalcin via cAMP/PKA and Ca^2+^/PKC signaling pathways [69–71]. Apart from these effects, PTH can interact with mediators between the kidney and bone, such as 1,25(OH)_2_D_3_, FGF-23 and calcium. The decline of renal function results in the deficiency of 1,25(OH)_2_D_3_ and subsequent decrease of serum calcium, which increase the synthesis and secretion of PTH and even the hyperplasia of parathyroid gland through VDR and CaSR/IP3 mediated signaling pathways, respectively [7, 72]. The PTH can inversely increase serum calcium by promoting its reabsorption by the kidney and release from bone; it can also increase 1,25(OH)_2_D_3_ levels by stimulating its production and inhibiting its degeneration in the kidney [73, 74]. Furthermore, elevated PTH can increase FGF-23 expression in bone either by activation of PKA and Wnt signaling pathways in osteoblasts or by promoting 1,25(OH)_2_D_3_ production [22, 75]. Meanwhile, the parathyroid gland is one of the target organs of FGF-23 and it can inhibit PTH synthesis either by cooperating with Klotho or via mitogen-activated protein kinases (MAPK) and calcineurin-mediated pathways independently [76, 77]. Therefore, there are several negative feedback loops between these factors, and their interactions play a very important role in the crosstalk between the kidney and bone.

## Conclusion

**Fig. 5:**
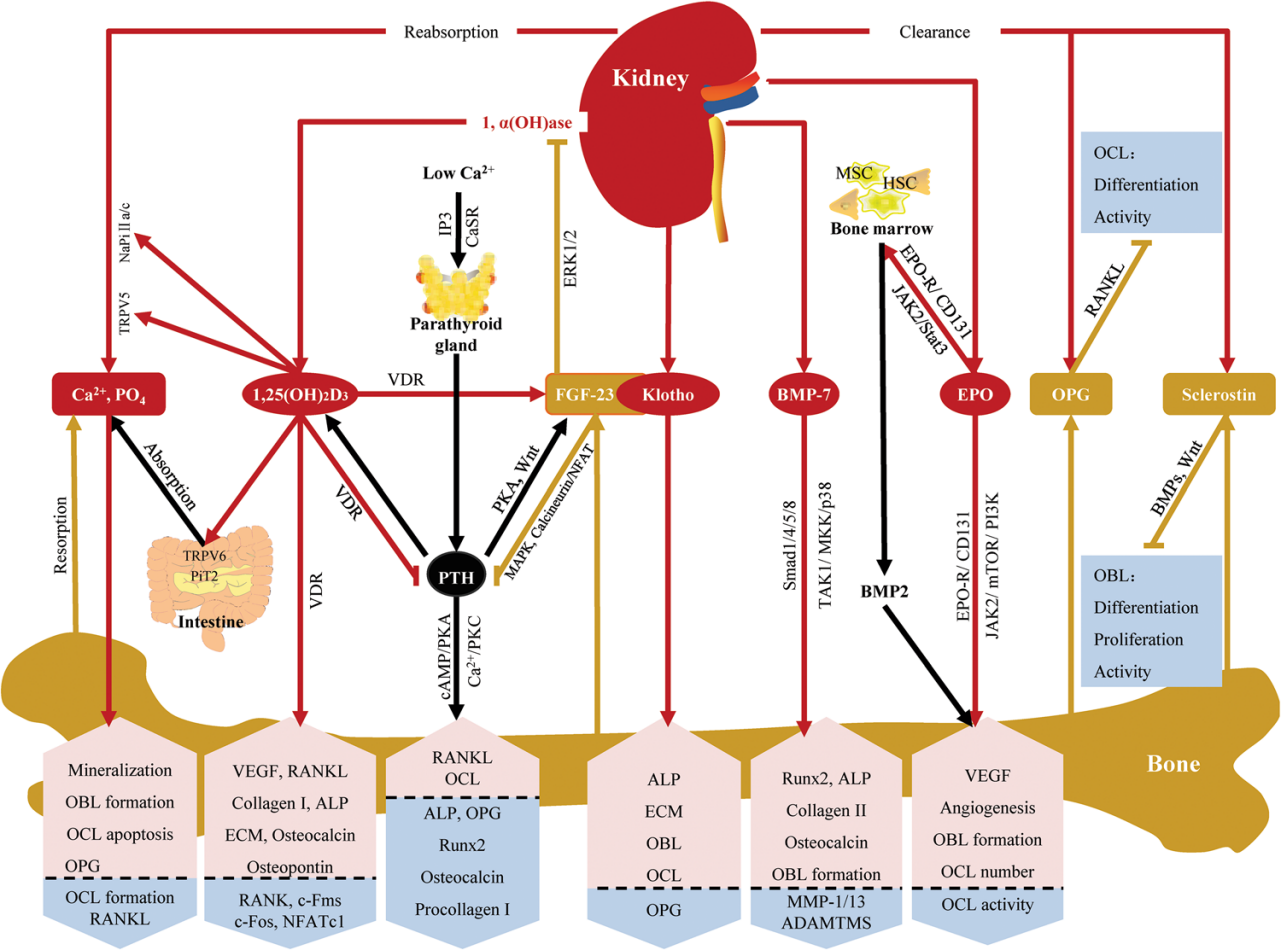
The kidney affects bone development, remodeling and repair by regulating calcium and phosphate homeostasis, producing cytokines and clearing bone regulators. (1) The kidney reabsorbs calcium and phosphate via TRPV6 and NaPi IIa/c. Calcium and phosphate are not only components of bone but also exert direct effects on bone cells via CaSR mediated signaling pathways. (2) The kidney synthesizes 1,25(OH)_2_D_3_, which can increase calcium and phosphate absorption and reabsorption by stabilizing their transporters. It can also exert direct effects on bone via VDR mediated signaling pathways and increase FGF-23 production in bone. (3) The kidney produces Klotho, which functions as a collaborator with bone-derived FGF-23 to decrease 1,25(OH)_2_D_3_ or exerts direct effects on bone independently. (4) The kidney produces BMP-7, which can exert direct effects on bone by activating Smad1/4/5/8 or TAK1/MKK/p38 signaling pathways. (5) The kidney synthesizes EPO, which has both direct effects on bone by activating JAK2, mTOR and PI3K signaling pathways and indirect effects through bone marrow. (6) OPG and sclerostin are bone-derived bone regulators. They are mainly cleared in the kidney. (7) PTH mediates the crosstalk between the kidney and bone. There are several feedback loops between PTH and serum calcium, 1,25(OH)_2_D_3_ and FGF-23. : produce or promote, : inhibit,  (*red arrow*) represents the effects of kidney,  (*yellow* *arrow*) represents the effects of bone,  (*black arrow*) represents the effects of others,  (*pink box with up arrow*) represents up-regulation or promotion,  (*blue box with down arrow*) represents down-regulation or inhibition, 1α(OH)ase: 1α-hydroxylase, ADAMTMS: a disintegrin and metalloproteinase with thrombospondin motifs, ALP: alkaline phosphatase, BMP: bone morphogenetic protein, Ca^2+^: calcium, CaSR: calcium-sensitive receptor, ECM: extracellular matrix, FGF: fibroblast growth factor, HSC: hematopoietic stem cell, MMP: metalloproteinase, MSC: mesenchymal stem cell, OBL: osteoblast, OCL: osteoclast, OPG: osteoprotegerin, PO_4_: phosphate, PTH: parathyroid hormone, RANKL: receptor activator of nuclear factor NF-κβ ligand, Runx2: runt-related gene 2, VDR: vitamin D receptor, VEGF: vascular endothelial growth factor

The kidney and bone are tightly coupled from early embryonic development, and the relationship between them is far more complex. The kidney affects bone development, remodeling and repair by regulating calcium and phosphate homeostasis, producing cytokines and clearing bone regulators (Fig. 5). As all of these actions have pleiotropic effects not only on bone but also on other tissues or organs, to fully understand their diverse impacts is difficult. However,it at least gives us some hints that kidney diseases should be taken into consideration when patients present with osteodystrophy and disturbances in bone and mineral metabolism. For some of these patients, treatment for renal dysfunction may accelerate their recovery. Finally, we hope that new discoveries of the roles played by renal cytokines in bone and an increased awareness of the underlying pathophysiologic mechanisms will pave the way for more individualized therapies.
